# Chitosan-Mediated Metabolic Regulation Alleviates Cold Damage and Enhances Quality in Dwarf Bananas

**DOI:** 10.3390/foods15081438

**Published:** 2026-04-20

**Authors:** Qun Zhang, Chunhua Liu, Miaomiao Su, Jia Song, Lehe Tan, Bingqiang Xu, Wenjiang Dong, Mingyue Wang, Daizhu Lyu

**Affiliations:** 1 Key Laboratory of Nutritional Quality and Health Benefits of Tropical Agricultural Products of Haikou City, Hainan Provincial Key Laboratory of Quality and Safety for Tropical Fruits and Vegetables, Key Laboratory of Quality and Safety Control for Subtropical Fruit and Vegetable, Ministry of Agriculture and Rural Affairs, Analysis and Test Center, Chinese Academy of Tropical Agricultural Sciences, Haikou 571101, China; zhangqun123@zju.edu.cn (Q.Z.); chunhualiu111@126.com (C.L.); summ1203@163.com (M.S.); jia668837@163.com (J.S.); tlh3687@163.com (L.T.); 2 Institute of Tropical Bioscience and Biotechnology, Chinese Academy of Tropical Agricultural Sciences, Haikou 571101, China; xubingqiang@itbb.org.cn; 3 Spice and Beverage Research Institute, Chinese Academy of Tropical Agricultural Sciences, Wanning 571533, China; dongwenjiang.123@163.com

**Keywords:** cold stress, dwarf banana, lipidomic, metabolomics, mitigating

## Abstract

Dwarf bananas are an important tropical fruit crop. They are particularly susceptible to cold stress, which often leads to quality deterioration. Although previous studies have examined the effects of cold stress on dwarf bananas, research on effective regulatory strategies and underlying mechanisms remains limited. This study investigates the mechanistic regulatory effects of chitosan (CTS) on cold stress in postharvest dwarf bananas, revealing that CTS mitigates cold-induced injury and improves fruit quality. Using an integrated approach of metabolomics, lipidomics, and enzyme activity assays, this study explored the potential mechanisms by which CTS alleviates chilling injury. Lipidomic results showed that CTS enhances cold tolerance by regulating the metabolism of glycerides, glycerophospholipids, linoleic acid, and linolenic acid. Metabolomics data further indicated that CTS increases the levels of amino acids, carbohydrates, and key substrates and intermediates of the tricarboxylic acid (TCA) cycle in cold-stressed dwarf bananas. Collectively, these effects enhance respiration, energy homeostasis, and antioxidant capacity, enabling dwarf bananas to better tolerate low-temperature stress.

## 1. Introduction

Dwarf banana (*Musa* ABB Pisang Awak ‘Guangfen No.1’) is an important variety within the Musa genus. It is known for its unique flavor, which is characterized by sweetness with a slight sour note, making it popular in the consumer market [1]. However, as a cold-sensitive fruit, the dwarf banana is highly susceptible to chilling injury (CI) due to cold waves and low-temperature storage in southern regions. CI manifests as peel browning or blackening, abnormal pulp ripening, and placental hardening, resulting in the loss of texture and flavor and ripening disorders [1]. These defects reduce its commercial value, limit market acceptance, and ultimately contribute to product loss and waste. Currently, traditional postharvest processing of bananas primarily focuses on preservation during transportation and ripening in the terminal market. There are no effective methods available to alleviate the cold damage that has already occurred or to restore the quality of the fruit. Therefore, identifying safe, healthy, and cost-effective exogenous substances to alleviate CI and improve flavor quality is essential for production sustainability. Research on cold storage of dwarf bananas remains limited, with most studies on cold resistance focusing on Cavendish bananas. Exogenous substances such as salicylic acid [2], arachidonic acid [3], melatonin [4], hydrogen sulfide [5,6], fibrin [7], calcium chloride [8], astragalus polysaccharides [9], and brassinosteroids have been shown to enhance cold stress resistance in Cavendish bananas and effectively reduce CI [10]. However, studies addressing CI in dwarf bananas are scarce and need further exploration.

Chitosan (CTS), also known as deacetyl chitin, chitosan amine, and chitosan polysaccharide, is a nontoxic, high-molecular-weight cationic polysaccharide widely applied in recent decades to regulate CI in fruit and vegetables [11]. CTS forms a protective film on the fruit surface that resists microbial invasion, prevents water loss, and preserves freshness [12]. It also reduces malondialdehyde (MDA) levels, decreases membrane lipid peroxidation, and improves antioxidant capacity [13,14]. In addition, CTS promotes nutrient accumulation and delays cellular senescence in fruits and vegetables [15,16]. CTS has been shown to alleviate cold damage in postharvest fruits, including tomatoes, cilantro, blueberries, and sweet cherries [6,17,18]. Therefore, in this study, we hypothesized that CTS mitigates cold-stress-induced damage in dwarf bananas.

Chilling injury is a major postharvest constraint that impairs the quality and commercial value of bananas. In the present study, 4 °C was selected as the storage temperature. This temperature was used to simulate the natural low-temperature stress that winter-harvested dwarf bananas typically encounter during harvest and transportation in major producing regions such as Guangxi and Yunnan, China. This study investigated the effects of CTS treatment on dwarf bananas exposed to cold stress, with a focus on reducing cold-induced damage and improving fruit quality. Furthermore, this study aimed to elucidate the mechanisms by which CTS exerts its effects. Few studies have investigated cold stress alleviation in dwarf bananas, and none have examined the impact of CTS on stress tolerance and fruit quality. Therefore, this study not only explores the mechanism of chilling injury in bananas under the low-temperature conditions prevalent in actual production practices but also provides a foundation for in-depth exploration of exogenous substance-mediated regulation of cold tolerance in dwarf bananas. Additionally, it offers novel insights into the potential application of CTS in postharvest cold storage and preservation, thereby laying a theoretical basis for enhancing the postharvest cold tolerance of winter-harvested bananas.

## 2. Materials and Methods

### 2.1. Chemicals and Reagents

Food-grade, acid-soluble chitosan (degree of deacetylation ≥ 90%) was supplied by Henan Zhigao Bio-technology Co., Ltd. (Zhengzhou, China). HPLC-grade ammonium acetate, zinc acetate, and anhydrous ethanol were obtained from Aladdin Reagent Co., Ltd. (Shanghai, China). The H_2_O_2_ assay kit was purchased from Beijing Solarbio Science and Technology Co., Ltd. (Beijing, China). Kits used for the determination of plant MDA, phospholipase D (PLD), and lipoxygenase (LOX) activities were provided by Suzhou Keming Biotechnology Co., Ltd. (Suzhou, China). Glucose, fructose, and sucrose standards (≥99.9% purity) were acquired from Merck Chemical Technology (Shanghai) Co., Ltd. (Shanghai, China). Analytical-grade potassium ferricyanide was supplied by Fuchen (Tianjin) Chemical Reagent Co., Ltd. (Tianjin, China).

### 2.2. Pre-Test Treatment

The test materials used for this experiment were dwarf banana (*Musa* ABB Pisang Awak ‘Guangfen No.1’) harvested in June 2023 from the Banana Demonstration Experimental Base of the Chinese Academy of Tropical Agricultural Sciences in Chengmai County, Hainan Province. A total of 300 green dwarf bananas, at the seven-ripe stage, of similar size, free from pests, diseases, and surface damage, were selected. The dwarf bananas were randomly assigned to the following three treatments: the control treatment, the CI treatment, and the CTS treatment, with 100 fruits in each treatment. After natural air-drying, all fruits were placed in polyethylene bags (200 mm × 50 mm, 0.03 mm). During storage, the relative humidity was maintained at 90% ± 5%. Dwarf bananas from the control treatment were soaked in 3 L of distilled water for 2 min, dried naturally, and then stored at 25 °C for 10 days. Fruits in the CI treatment were soaked in 3 L of distilled water for 2 min, dried naturally, and stored at 4 °C for 4 days, followed by storage at 25 °C for 6 days. Fruits in the CTS treatment were soaked in 3 L of 1% CTS solution for 2 min, dried naturally, placed in polyethylene bags, stored at 4 °C for 4 days, and then transferred to 25 °C for 6 days. To ensure experimental reliability and independence, six biological replicates were established, with each replicate consisting of three individual fruits. Throughout the entire experiment, all replicates were subjected to independent treatment, sampling, parameter determination, and data analysis. The samples were collected once the peel turned bright yellow at room temperature. The pulp was sliced, rapidly frozen in liquid nitrogen, and stored at −80 °C for physiological and biochemical analyses. The cold damage index was determined according to methods described previously [19,20], as shown in Supplementary Section S1.

### 2.3. Determination of H_2_O_2_ and MDA Contents and PLD and LOX Activities

The H_2_O_2_ content was determined in accordance with the instructions of the kit from Beijing Solarbio Biotechnology Co., Ltd. MDA content, PLD, and LOX activities were determined according to the instructions of Suzhou Keming Biotechnology Co., Ltd.; the detailed procedures are presented in Supplementary Sections S2–S5.

### 2.4. Lipidomic Analysis

For lipidomic analysis, the pulp of dwarf bananas was ground to a fine powder in liquid nitrogen, and 100 mg of the sample was transferred to an EP tube containing 400 μL of water and vortexed for 30 s. An extraction solution (960 μL, MTBE: methanol = 5:1, *v*/*v*) was then added, the mixture was vortexed, sonicated in an ice bath for 15 min, incubated at −20 °C for 1 h, and then centrifuged at 4 °C, 10,000 rpm for 15 min. A total of 760 μL of the supernatant was extracted, dried under nitrogen gas, and reconstituted in 200 μL of dichloromethane: methanol (1:1, *v*/*v*). The mixture was vortexed, sonicated in an ice bath for 10 min, centrifuged at 13,000 rpm for 15 min, and 180 μL of the supernatant was transferred into autosampler vials. Quality control (QC) samples were prepared by pooling 20 μL of each sample, with QC injections performed every 10 samples to monitor instrument stability. Lipid analysis was performed using UPLC-MS/MS in both positive and negative modes (Supplementary Section S6).

### 2.5. Metabolomics Analysis

The dwarf banana pulp was ground into a fine powder in liquid nitrogen, and 100 mg of the sample was transferred to a centrifuge tube containing 500 μL of 80% aqueous methanol supplemented with 0.1% formic acid. The mixture was vortexed for 30 s, incubated in an ice bath for 5 min, and then centrifuged at 4 °C, 13,000 rpm for 10 min. The supernatant was diluted to 60% methanol with ultrapure water, filtered through a 0.22 μm filter membrane, and transferred into an autosampler injection vial. QC samples were prepared by pooling 10 μL of each supernatant, and a blank sample was prepared using 60% methanol supplemented with 0.1% formic acid. Metabolomics profiling was performed using UPLC-Q-Exactive-MS/MS in both positive and negative modes (Supplementary Section S7).

### 2.6. Determination of Glucose, Fructose, and Sucrose

Dwarf banana samples (5 g) were weighed into a 100 mL volumetric flask and dissolved in approximately 50 mL of ultrapure water, to which 5 mL each of zinc acetate and potassium ferricyanide solutions were added. Water was added up to the mark, and the mixture was magnetically stirred or sonicated for 30 min, filtered through dry filter paper, with the initial filtrate discarded. The subsequent filtrate was passed through a 0.45 μm microporous filter membrane or centrifuged, and the supernatant was collected for analysis. Different concentrations of glucose, fructose, and sucrose standards were prepared (10.00, 5.00, 2.50, 1.00, 0.50, 0.25 mg/mL) using ultrapure water and quantified by the external standard method via liquid chromatography (Supplementary Section S8).

### 2.7. Statistical Analysis

Bar charts, violin plots, PCA (principal component analysis) plots, clustering heatmaps, bubble plots, volcano plots, and pie charts were generated using GraphPad Prism 8. *p* values (*n* = 6) were calculated using an independent samples *t*-test in IBM SPSS 25. Multiple testing correction was performed using the Benjamini–Hochberg false discovery rate (FDR) approach. Differential lipids were identified with fold change (FC) > 2 or FC < 0.5 and FDR-adjusted *q* values < 0.05. Differential metabolites were identified with |log_2_FC| > 0.57 and FDR-adjusted *q* values < 0.05. Both differential lipids (DILs) and differential metabolites (DIMs) were further subjected to KEGG pathway enrichment analysis using the MetaboAnalyst website (https://www.metaboanalyst.ca/).

## 3. Results

### 3.1. CTS Effect on the Quality of the Dwarf Banana

**Figure 1:**
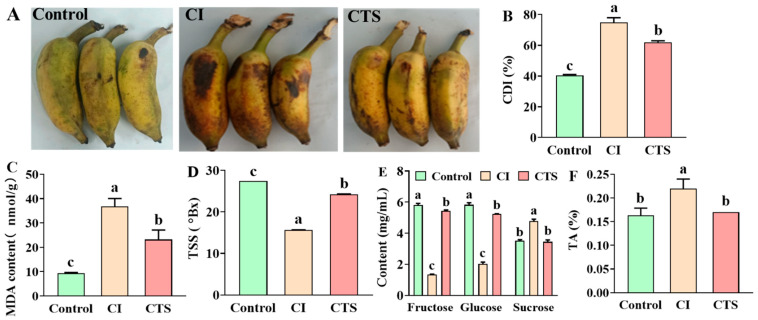
The study observed changes in (**A**) phenotype, (**B**) CDI, (**C**) MDA content, (**D**) TSS, (**E**) glucose, fructose, and sucrose content, and (**F**) TA, among the three treatments: Control, CI, and CTS. The treatments were as follows: Control (soaked in distilled water and stored at 25 °C for 10 days); CI (soaked in distilled water, stored at 4 °C for 4 days, followed by storage at 25 °C for 6 days); CTS (soaked in 1% CTS solution, stored at 4 °C for 4 days, followed by storage at 25 °C for 6 days). Data are presented as the mean ± SD (*n* = 6). Bars indicate standard errors. Different letters indicate significant differences (*p* < 0.05). Abbreviations: CDI, cold damage index; MDA, malondialdehyde; TSS, total soluble solids; TA, titratable acid; SD, standard deviation.

The effects of CTS on the appearance and flavor quality of dwarf bananas stored at 4 °C were assessed using the cold damage index (CDI), MDA content, total soluble solids (TSS), monosaccharide content, and titratable acidity (TA) of the dwarf bananas. Significant differences were observed among the three treatments: control, CI, and CTS (Figure 1). CTS treatment under cold stress reduced black peel spots, partially restored ripening behavior, and lowered the CDI to 64% (Figure 1A,B). Compared with the control, CI-treated bananas stored at 4 °C exhibited dark brown peel spots, inhibited ripening, hard texture, and a CDI of 73%. MDA content was reduced from 36.80 nmol/g to 23.08 nmol/g after CTS treatment (Figure 1C). These results indicate that CTS alleviated cold damage symptoms in dwarf bananas by reducing both CDI and lipid peroxidation, reflecting the protective effect of CTS on membrane integrity under cold stress conditions.

Compared to the control, CI treatment significantly decreased TSS in bananas (Figure 1D). The TSS of the CI treatment was 15.6 °Bx, equivalent to 56.8% of the control level. However, CTS treatment increased TSS (24.3 °Bx) under cold stress, thereby improving sweetness. Monosaccharide analysis showed that the fructose and glucose concentrations in the CTS treatment were 5.44 mg/mL and 3.45 mg/mL, respectively (Figure 1E). In contrast, the fructose and glucose concentrations in the CI treatment were 1.34 mg/mL and 2.03 mg/mL, respectively. Compared to the CI treatment, CTS treatment significantly increased fructose and glucose levels under cold stress and reduced sucrose content. Given that fructose is 1.9 times sweeter than sucrose and 3.7 times sweeter than glucose [21], its increase plays a key role in maintaining banana sweetness under cold stress. Under abiotic stresses such as drought and flooding, plants break down starch into monosaccharides, including glucose and fructose, which accumulate to provide energy for stress responses [22,23]. Glucose, as a central carbohydrate metabolite, is positively correlated with cold tolerance [24], and higher glucose content improves frost tolerance in loquat fruit [25]. In this study, CTS treatment maintained normal respiratory metabolism during the yellow ripening phase through glucose and fructose accumulation, thus preserving sweetness and enhancing cold tolerance.

The TA content in the CI treatment was 34.7% higher than that in the control (Figure 1F). Following CTS treatment, TA significantly decreased compared with the CI treatment and showed no significant difference from the control. These findings suggest that cold stress increases the acidity of dwarf bananas, which may negatively impact their sensory taste quality. CTS treatment under cold stress attenuated the increase in TA induced by cold stress. Pagno et al. [18] reported that cold storage treatment significantly increased the TA of olive fruit, affecting their taste. Treatment at 0 °C significantly increased TA in kiwifruit and reduced TSS [26]. Oligochitosan treatment significantly reduced TA compared with control treatments in apricot fruit [27], and exogenous substances such as arginine and phenylalanine also reduce TA in fruit [28], consistent with our results.

### 3.2. CTS Effect on Hardiness of the Dwarf Banana and Lipid Metabolism

**Figure 2:**
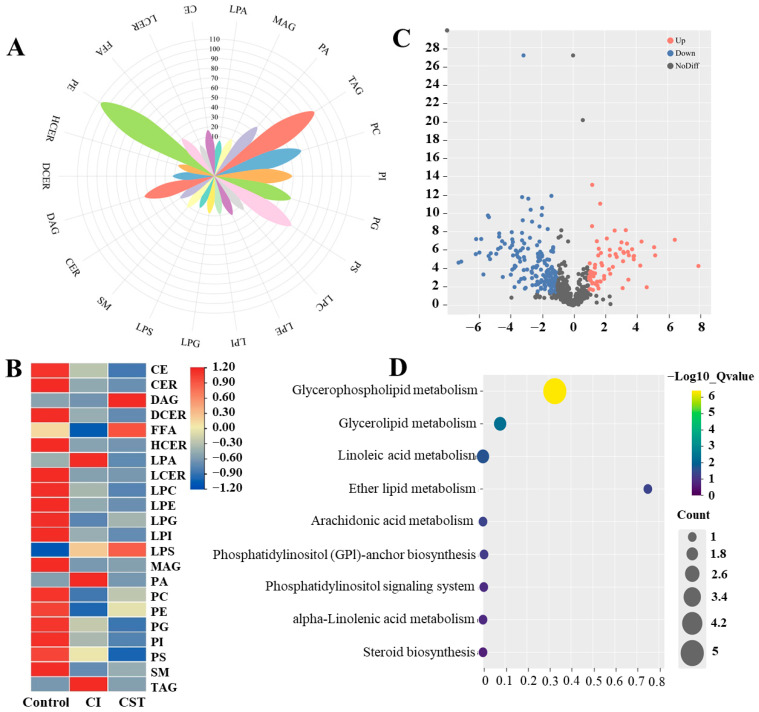
Petal plots of (**A**) lipid subclasses, (**B**) heat map of differential lipids, (**C**) volcano map of differential lipids, and (**D**) bubble map of KEGG enrichment analysis of differential lipids. Abbreviations: CE, cholesteryl esters; CER, ceramides; DAG, diacylglycerols; DCER, dihydroceramides; FFA, free fatty acids; HCER, hexosylceramides; LPA, lysophosphatidic acid; LCER, lactosylceramides; LPC, lysophosphatidylcholine; LPE, lysophosphatidylethanolamine; LPG, lysophosphatidylglycerol; LPI, lysophosphatidylinositol; LPS, lysophosphatidylserine; MAG, monoacylglycerols; PAG, phosphatidic acid; PC, phosphatidylcholines; PE, phosphatidylethanolamines; PG, phosphatidylglycerols; PI, phosphatidylinositols; PS, phosphatidylserines; SM, sphingomyelins; TAG, triacylglycerols.

Low-temperature stress directly affects the synthesis and dynamics of plant lipids. The cell membrane is considered the primary site of damage under cold stress, and maintaining membrane stability and fluidity is crucial for plant survival under adverse conditions [29]. Lipids, as fundamental structural components of cell membranes, play key roles in energy retention and supply as well as in signal transduction within biological systems [30]. This study employed LC-MS/MS to analyze lipids in the different treatments, identifying 655 lipid species across 22 subclasses within five major categories: glycerolipids (167), glycerophospholipids (369), lysolipids (55), sphingolipids (47), and sterols (17) (Figure 2A). The clustering heat maps revealed significant differences between control and treatment samples (Figure 2B), indicating that CTS significantly altered lipid metabolism in cold-stressed bananas compared to the CI treatment, thereby reshaping the lipid composition under cold stress.

Univariate analysis (FC ≥ 2 or FC ≤ 0.5 and *p* < 0.05) identified 327 DILs between the CI treatment and the CTS treatment (Figure 2C). Among these, 104 lipids were upregulated, primarily DAG, FFA, LPG, LPS, MAG, PC, PE, and SM, while 223 lipids were downregulated, mainly CE, LPA, LPC, PA, PI, PS, and TAG subclasses. These results suggest that CTS modulates the accumulation of specific lipid molecules in dwarf bananas under cold stress. KEGG pathway enrichment analysis of the 327 DILs revealed significant enrichment in three membrane lipid metabolic pathways (*p* < 0.05), namely glycerophospholipid metabolism, glycerolipid metabolism, and linoleic and α-linolenic acid metabolism (Figure 2D). This indicates that membrane lipid metabolism is potentially involved in CTS-regulated cold stress tolerance in postharvest dwarf bananas, rather than directly mediating postharvest cold storage recovery.

**Figure 3:**
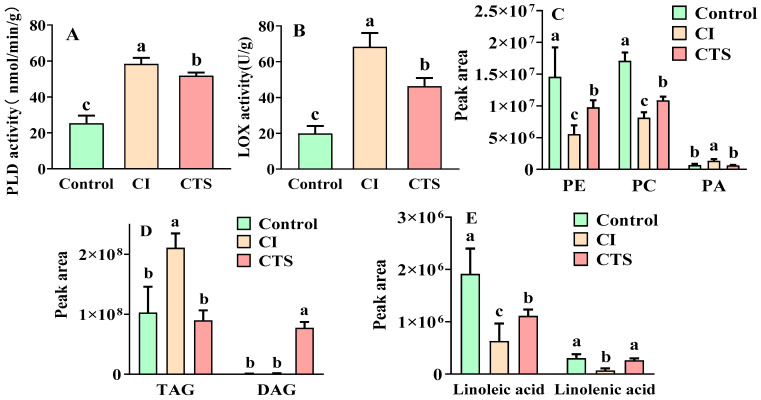
Petal plots of (**A**) PLD activity; (**B**) LOX activity; (**C**) PE, PC, and PA contents; (**D**) TAG and DAG contents; and (**E**) linoleic acid content changes, among the three treatments: Control, CI, and CTS. The treatments were as follows: Control (soaked in distilled water and stored at 25 °C for 10 days); CI (soaked in distilled water, stored at 4 °C for 4 days, followed by storage at 25 °C for 6 days); CTS (soaked in 1% CTS solution, stored at 4 °C for 4 days, followed by storage at 25 °C for 6 days). Data are presented as the mean ± SD (*n* = 6). Different letters indicated significant differences (*p* < 0.05). Abbreviations: PLD, phospholipid-degrading enzyme; LOX, lipoxygenase; PA, phosphatidic acid; PC, phosphatidylcholine; PE, phosphatidylethanolamine; TAG, triacylglycerol; DAG, diacylglycerol; SD, standard deviation.

Analysis of proteins involved in glycerophospholipid metabolism revealed that CTS affects key membrane lipids, including PC, PE, and PA. PC and PE are important structural components of plant membranes, with their levels significantly influencing membrane stability and integrity. PA, an important signaling molecule in cell membranes, can disrupt normal physiological functions of plants if its content rises excessively. PLD and LOX are key enzymes in fruits that also play important roles in plants’ responses to cold stress. Increased LOX activity promotes peroxidation of membrane lipids, destabilizing cells, whereas elevated PLD activity accelerates degradation of structural membrane lipids, PC and PE, converting them into PA [31,32]. Therefore, we analyzed changes in PC, PE, and PA under CTS treatment alongside PLD and LOX activity indicators (Figure 3A–C) to explore the potential regulatory mechanism by which CTS alleviates cold stress in bananas. These findings suggested that cold stress may upregulate LOX and PLD activities in dwarf bananas, which is potentially associated with the accelerated degradation of PC and PE, the subsequent reduction in their contents, and the accumulation of the signaling lipid PA. Consequently, MDA, a secondary product of lipid peroxidation, reached 36.80 nmol/g in the CI treatment, causing damage to the banana fruit. CTS treatment decreased MDA content to 23.18 nmol/g and LOX activity by 22.08 U/g, reducing membrane lipid peroxidation. CTS also increased PC and PE levels while reducing PA content. As major cytoplasmic membrane components, PC and PE maintain cellular stability, store and release energy, and preserve cellular structure and function. CTS downregulated seven PA species, including PA (14:0), PA (16:0), and PA (18:0), alleviating the toxic effects of excessive PA accumulation. The role of exogenous substances in enhancing fruit cold resistance through lipid remodeling has been demonstrated [29,33,34]. It has been reported that 0.5 mmol/L melatonin treatment of “Fei Fei” mango reduced PLD and LOX activities, decreased relative electrolyte leakage and MDA content, increased PC and PE levels, preserved membrane structure, and activated downstream signaling pathways, thereby improving cold tolerance [30]. Similarly, dipping ‘Zaosu’ pears in 100 mg/L ethyl-Nα-lauroyl-L-arginate hydrochloride resulted in the down-regulation of PLD and LOX activities, inhibited phospholipid degradation, and thereby delayed senescence during storage at 21 °C [35].

Analysis of the glycerolipid metabolic pathway revealed that CTS treatment significantly increased DAG content compared with the CI treatment (Figure 3D). Previous research has reported that exogenous DAG enhances cold tolerance in Arabidopsis SAG101, EDS1, and PAD4 mutants [36]. In the glycerolipid metabolic pathway, CTS primarily alleviated cold stress responses in bananas by increasing DAG levels, thereby improving cold tolerance. Furthermore, CTS significantly increased linoleic acid and α-linolenic acid contents by 1.6 and 3.4 times (Figure 3E), respectively. These results indicate that under CTS treatment, elevated unsaturated fatty acid levels and reduced acyl chain packing density. This observation suggests that the elevated levels of unsaturated fatty acids induced by CTS may strengthen the bilayer structure of banana cell membranes, thereby potentially enhancing membrane stability and integrity, reducing cellular damage [37], and contributing to the improvement of cold tolerance in dwarf bananas under cold stress conditions.

### 3.3. CTS Effect on Hardiness of the Dwarf Banana and Amino Acid and Carbohydrate Metabolic Pathways

**Figure 4:**
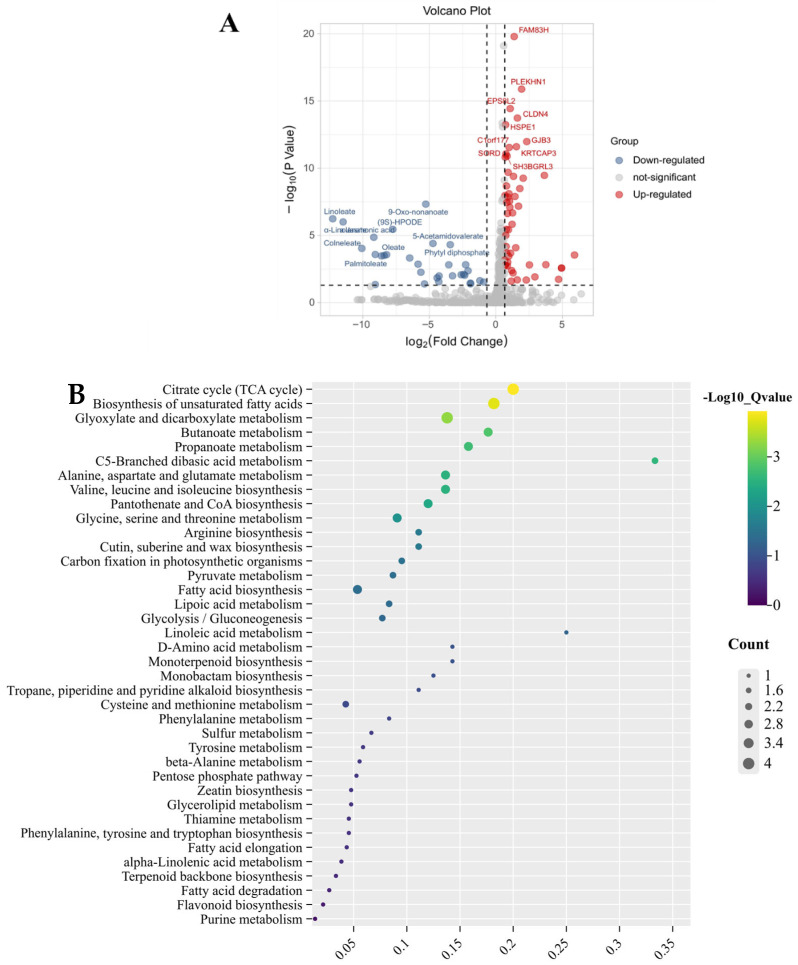
(**A**) Volcanic maps of differential metabolites; (**B**) bubble map of KEGG enrichment analysis of differential metabolites of the CI and CTS treatments. The treatments were as follows: CI (soaked in distilled water, stored at 4 °C for 4 days, followed by storage at 25 °C for 6 days); CTS (soaked in 1% CTS solution, stored at 4 °C for 4 days, followed by storage at 25 °C for 6 days).

Untargeted metabolomics detected 383 metabolites in dwarf bananas under ESI positive and negative ion modes, including 164 organic acids, 132 other metabolites, 34 esters and derivatives, 30 amino acids, and 23 sugars. DIMs between the CI and CTS treatments were screened using statistical analyses (Log_2_FC > 0.57 or Log_2_FC < −0.57 and *p* < 0.05). Compared with the CI treatment, CTS treatment resulted in 73 downregulated and 43 upregulated metabolites (Figure 4A). Upregulated metabolites included sugars such as glucose, fructose, and α, α-trehalose, as well as organic acids such as pyruvate, oxaloacetate, and fumarate. Downregulated metabolites included organic acids such as (S)-malate, jasmonic acid, and (R)-pantothenic acid. Pathway enrichment analysis identified 37 metabolic pathways affected by CTS (Figure 4B), with notable enrichment in amino acid and carbohydrate metabolism. The primary metabolic variations in the amino acid pathway were associated with alanine, aspartate, and glutamate metabolism. Lipoic acid metabolism was also significantly affected by CTS treatment. Similarly, in carbohydrate metabolism, notable differences were observed in butyric acid metabolism and C5-branched dibasic acid metabolism.

**Figure 5:**
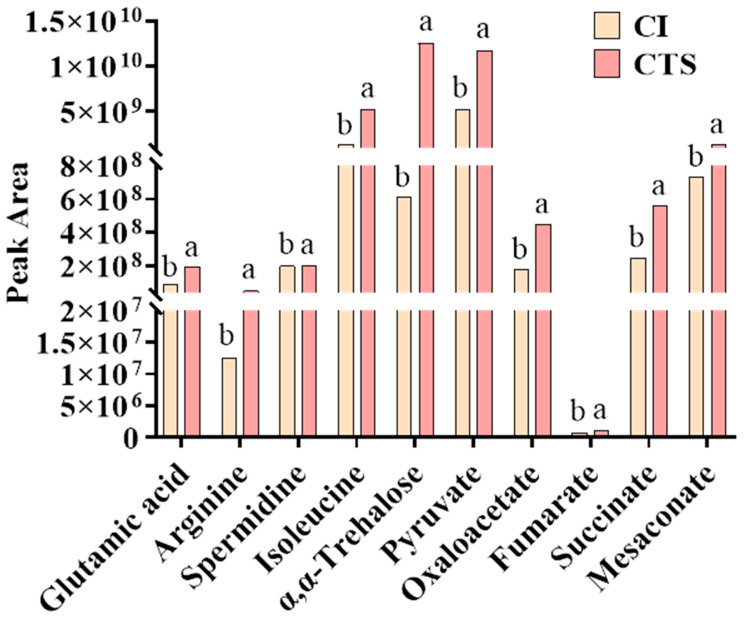
Significant changes in some DIMs in the CI and CTS treatments. The treatments were as follows: CI (soaked in distilled water, stored at 4 °C for 4 days, followed by storage at 25 °C for 6 days); CTS (soaked in 1% CTS solution, stored at 4 °C for 4 days, followed by storage at 25 °C for 6 days). Data are presented as the mean ± SD (*n* = 6). Different letters indicated significant differences (*p* < 0.05). Abbreviations: DIMs, differential metabolites; SD, standard deviation.

Metabolite analysis revealed that CTS treatment significantly increased the contents of glutamate, spermidine, and isoleucine in dwarf bananas (Figure 5, Supplementary Section S9). Glutamate, an acidic amino acid, enhances the antioxidant capacity either as a proton donor paired with a lone pair of electrons or indirectly by increasing antioxidant enzyme activity [38]. It also serves as a precursor for the non-enzymatic antioxidant glutathione [39]. Therefore, elevated glutamate levels may increase the antioxidant capacity of dwarf bananas, reducing cellular damage and enhancing stress resistance. CTS treatment also significantly increased spermidine levels. The polycationic nature of spermidine enables strong binding to cell membranes and cell walls [40]. Increased spermidine helps maintain membrane integrity and strengthen cell wall stability under cold stress, contributing to enhanced cold tolerance. CTS also increased the isoleucine content. Isoleucine has been reported to regulate plant stress tolerance via the respiratory system [41]. Meanwhile, the carbon skeleton of branched-chain amino acids can be converted into precursors or intermediates of the tricarboxylic acid (TCA) cycle for ATP production, supporting plant energy homeostasis [42]. It is hypothesized that higher isoleucine levels may contribute to the improvement of cold tolerance in dwarf bananas, potentially by regulating respiratory metabolism and energy homeostasis under cold stress conditions.

Many studies have shown that the energy status in fruits and vegetables directly affects membrane lipid remodeling and cell membrane stability. In this study, CTS treatment significantly elevated α,α-trehalose, 2-deoxy-L-sucrose, and D-glucopyranose levels in dwarf bananas (Figure 5, Supplementary Section S9), which are important substrates for sugar metabolism. Sugars serve not only as the primary energy source in plants but also as essential components of structural substances [43]. Therefore, we hypothesize that chitosan may stabilize cell membrane structures and alleviate low-temperature damage by regulating sugar metabolism in dwarf bananas and improving cellular energy status. Meanwhile, CTS treatment significantly upregulated the contents of five organic acids, including pyruvic acid, oxaloacetic acid, fumaric acid, succinic acid, and mesaconic acid, all of which are key substrates and important intermediates of the TCA cycle. The TCA cycle, a key respiratory metabolism pathway, regulates the ability of fruits and vegetables to resist external stresses by utilizing metabolic substrates [42,44,45]. In this study, CTS treatment induced significant glucose accumulation in dwarf bananas. This glucose was phosphorylated to glucose-6-phosphate by hexokinase, and then funneled through a cascade of enzymatic transformations, ultimately producing pyruvate. Pyruvate, a vital carbohydrate and primary entry substrate for the TCA cycle, promoted the enhanced production of oxaloacetic acid, fumaric acid, succinic acid, and mesaconate. Our results indicate that CTS treatment may promote the accumulation of key intermediates in the TCA cycle, including pyruvic acid, oxaloacetic acid, fumaric acid, succinic acid, and mesaconic acid, in cold-stressed dwarf bananas. This metabolic alteration may potentially support the partial operation of the TCA cycle and maintain cellular energy homeostasis under low-temperature stress, which may contribute to the alleviation of chilling injury symptoms in postharvest dwarf bananas. However, to further clarify how CTS enhances cold resistance and recovery in bananas by regulating the metabolic network, additional time-series sampling is required, along with relevant physiological indicators and molecular biological analyses for verification and mechanistic investigation.

## 4. Conclusions

This study employed a multidimensional approach that integrates lipidomics and metabolomics to explore the mechanistic basis of the exogenous substance CTS on regulating cold tolerance of postharvest dwarf bananas. The findings are as follows: CTS can significantly increase the content of key membrane lipids in dwarf bananas, providing a material basis for the stability of cell membrane structure and function, thus potentially contributing to their cold resistance. Simultaneously, CTS systematically regulates the metabolic network of dwarf bananas and upregulates amino acid metabolic pathways to support cellular energy supply and metabolic balance; it promotes sugar metabolism processes to ensure energy supply and elevates the levels of intermediate metabolites such as pyruvate, oxaloacetate, and fumarate, which may help strengthen energy metabolism and material circulation in the tricarboxylic acid cycle metabolic pathway. Therefore, it is hypothesized that CTS may establish a complex metabolic network for dwarf bananas to cope with cold stress through the coordinated regulation of lipid metabolism, sugar metabolism, and amino acid metabolism, leading to a systematic enhancement of cold resistance from the perspectives of respiration, plant energy homeostasis, and antioxidant defense. This study provides a theoretical basis for understanding the mechanisms by which exogenous substances regulate the cold resistance of dwarf bananas and offers new insights for optimizing postharvest cold storage technology, supporting further in-depth exploration.

## Data Availability

The data presented in this study are available on request from the corresponding author.

## Supplementary Materials

The following supporting information can be downloaded at https://www.mdpi.com/article/10.3390/foods15081438/s1. Supplementary Section S1: Determination of the cold damage index; Supplementary Section S2: Method for the determination of hydrogen peroxide (H_2_O_2_) content; Supplementary Section S3: Method for the determination of malondialdehyde (MDA) content; Supplementary Section S4: Phospholipid-degrading enzyme (PLD) assay; Supplementary Section S5: Lipoxygenase (LOX) assay; Supplementary Section S6: Lipidomics analysis (chromatographic conditions and elution procedures); Supplementary Section S7: Metabolomics analysis (chromatographic conditions and elution procedures); Supplementary Section S8: Determination of glucose, fructose, sucrose (chromatographic methods). Supplementary Section S9: Some differential metabolites (DIMs) in the Control, CI and CTS treatments. Table S1. The degree of the five-level cold damage. Table S2. Reagent components, specifications and storage conditions for the determination of hydrogen peroxide (H_2_O_2_) content. Table S3. H_2_O_2_ content extraction solution scheme and centrifugation conditions. Table S4. PLD extraction solution scheme and centrifugation conditions. Reference [46] is cited in the Supplementary Materials.
