# Supplementary material for: Chitosan-Mediated Metabolic Regulation Alleviates Cold Damage and Enhances Quality in Dwarf Bananas
Source: Foods. 2026 Apr 20;15(8):1438. doi: 10.3390/foods15081438 (PMC13116575; doi:10.3390/foods15081438)
Supplement: Supplementary file 1 [file foods-15-01438-s001.zip › foods-4201113-supplementary.pdf]

Section S1. Determination of the cold damage index.
Section S2. Method for the determination of hydrogen peroxide (H_2O_2) content.
Section S3. Method for the determination of malondialdehyde (MDA) content.
Section S4. Phospholipid-degrading enzyme (PLD) assay.
Section S5. Lipoxygenase (LOX) assay.
Section S6. Lipidomics analysis (Chromatographic conditions and elution procedures).
Section S7. Section Metabolomics analysis (Chromatographic conditions and elution procedures).
Section S8. Determination of glucose, fructose, sucrose (Chromatographic methods).
Section S9. Some DIMs in the Control, CI and CTS treatments.
Table S1. The degree of the five-level cold damage.
Table S2. Reagent components, specifications and storage conditions for the determination of hydrogen peroxide (H_2O_2) content.
Table S3. H_2O_2 content extraction solution scheme and centrifugation conditions.
Table S4. PLD extraction solution scheme and centrifugation conditions.
References

Section S1. Determination of cold damage index:

The cold damage index was determined according to Yang [1]**.** The degree of cold damage was evaluated in terms of surface colour and browning and was classified into 5 levels (**Table S1**). All evaluations were performed by three independent trained assessors, and assessors were blinded to treatment groups during scoring to minimize observer bias.

Cold damage index = ∑ (cold damage level × number of fruits at this stage) / (4 × total number of fruits)

Table S1. The degree of the five-level cold damage

**Chilling injury level**	**Evaluation criterion**
0 level	No signs of cold damage
Level 1	Cold damage 0 ~ <25% of total pericarp area
Level 2	Cold damage to 25% ~ <50% per cent of the total pericarp area
Level 3	Cold damage to 50% ~ <75% per cent of the total pericarp area
Level 4	Cold damage to 75% ~ <100% per cent of the total pericarp area

Section S2. Method for the determination of hydrogen peroxide (H_2O_2) content:

Reagent components, specifications and storage conditions for the determination of hydrogen peroxide (H_2O_2) content prepared followed by **Table S2**. Specific steps: The spectrophotometer was preheated for more than 30 min, the wavelength was adjusted to 415 nm, and distilled water was used to adjust the zero. Weigh about 0.1 g of the powdered plantain sample, add 1ml of extraction solution, grind thoroughly on ice, centrifuge at 8000 g 4 ℃ for 10 min, take the supernatant. Add the following reagents in order in an EP tube, according to **Table S3**. After adding reagent IV to dissolve the precipitate, it was allowed to stand at room temperature for 5 min, and 200 μL was transferred to a micro glass cuvette to determine the absorbance value at 415 nm. In 1 ml of the system, 0.01 units of catalytic absorbance value change per minute per gram of sample in 25 °C is one unit of enzyme activity. The calculation formula is as follows:

ΔA assay = A assay tube - A blank tube;

ΔA standard = A standard tube - A blank tube;

H_2O_2 Content (μmol/g) = ΔA assay ÷ (ΔA standard ÷ C standard solution) × V sample ÷ (V sample ÷ V extraction × W) = ΔA assay ÷ ΔA standard ÷ W;

V sample: volume of the sample added, 0.25 mL;

V extraction: volume used in the extraction process, 1 mL;

W: tissue mass, g.

Table S2. Reagent components, specifications and storage conditions for the determination of hydrogen peroxide (H_2O_2) content

Reagent name	Specification	Preservation condition
Reagent I	Liquid 100 mL×1 bottle	4 °C storage
Reagent II	Powder×1 bottle	4 °C storage
Reagent III	Liquid 6 mL×1	4 °C storage
Reagent IV	Liquid 30 mL×1 bottle	4 °C storage
Standard product	Liquid 1 mL × 1	4 °C storage

Preparation of solutions: 1. Reagent I: acetone is self-contained; 2. Reagent two: before use, add 3 mL of concentrated hydrochloric acid to fully dissolve standby, the use of inexhaustible reagents stored at 4 ℃; 3. Standards: 1 mmol/mL H_2O_2 standard solution.

Table S3. H_2O_2 content extraction solution scheme and centrifugation conditions

**Reagent name (μL)**	**Measuring tube**	**Standard tube**	**Blank tube**
Blank tube	250		
Standard solution		250	
Reagent I			250
Reagent II	25	25	25
Reagent III	50	50	50
4000g, centrifuge at room temperature for 10 min, discard the supernatant, leave the precipitate (can be washed with acetone 3-5 times to wash away the plant colouring first)			

Reagent IV	250	250	250

Section S3. Method for the determination of malondialdehyde (MDA) content:

Weigh about 0.1 g of powdered plantain sample, add 1 mL of extraction solution, grind thoroughly on ice, centrifuge at 8000 g at 4 ℃ for 10 min, take the supernatant, aspirate 0.6 mL of reagent I in a 1.5 mL centrifuge tube, then add 0.2 mL of centrifuged sample, mix well. Keep warm in 95 ℃ water baths for 30 min (cover tightly to prevent water loss), cool in ice bath, centrifuge at 10000 g at 25 ℃ for 10 min, take up the supernatant in 1 mL glass cuvette, determine the absorbance at 532 nm and 600 nm, recorded as A532 and A600, △A= A532-A600.

One unit of enzyme activity is defined as a change of 0.01 units of catalytic absorbance value per minute per gram of sample in a system of 1 mL at 25°C.

MDA content (nmol/g) = [(ΔA × V anti total ÷ (ε × d) × 109 ÷ (W × V sample ÷ V sample total) = 25.8 × ΔA ÷ W.

ε is the molar extinction coefficient of malondialdehyde, 155 × 103L/mol/cm;

d. cuvette aperture, 1 cm;

V sample: volume of sample added, 0.2 mL;

V sample total, volume of extract added, 1 mL;

W is the mass of sample, g.

Section S4. Phospholipid-degrading enzyme (PLD) assay:

The spectrophotometer was preheated for more than 30 min, the wavelength was adjusted to 500 nm, and distilled water was used for zero adjustment. Weigh about 0.1 g of the powdered plantain sample, add 1 mL of the extraction solution, grind thoroughly on ice, centrifuge at 10000 g 4 ℃ for 5 min, discard the supernatant, take the precipitate and dissolve it in 1mL of reagent I. Add the following reagents in order in the EP tube (**Table S4**).

Table S4. PLD extraction solution scheme and centrifugation conditions

<table>
<tr><th>Reagent name (μL)</th><th>Measuring tube</th><th>Standard tube</th><th>Blank tube</th></tr>
<tr><td>Reagent I</td><td>20</td><td></td><td></td></tr>
<tr><td>Reagent II</td><td>30</td><td>30</td><td>30</td></tr>
<tr><td>Standard product</td><td></td><td>20</td><td></td></tr>
<tr><td>Specimens</td><td></td><td></td><td></td></tr>
<tr><td>Reagent III</td><td>10</td><td>10</td><td>10</td></tr>
<tr><td colspan="4">Mix well, react at 30℃ for 30 min, boil water bath for 1min, open the lid and cool naturally for 2min.</td></tr>
<tr><td>Reagent IV</td><td>140</td><td>140</td><td>140</td></tr>
<tr><td colspan="4">The reaction was carried out at 30 °C for 30 min, the blank tube was adjusted to zero, and the absorbance at 500 nm was measured and recorded as A standard tube and A assay tube respectively.</td></tr>
</table>

PLD activity (nmol/min /g) = A tube/A standard tube × C standard ÷ W ÷ T = 0.017 × A tube/A standard tube ÷ W

C Standard: standard concentration, 500 nmol/L; W: sample mass, g/mL; T: reaction time, 30 min.

Section S5. Lipoxygenase (LOX) assay:

Preheat the spectrophotometer or enzyme labeler for more than 30 min, adjust the wavelength to 234 nm, and adjust the zero with distilled water. Weigh about 0.1 g of the powdered plantain sample, add 1mL of the extract, grind it thoroughly on ice, centrifuge at 16000 g for 20 min at 4 ℃, take the supernatant and put it on ice to be measured. Preheat the photometer for more than 30 min, adjust the wavelength to 234 nm, and adjust the zero with distilled water. Control tube: add 100 μL of distilled water, 800 mL of reagent 1 and 100 μL of reagent 2 into 1 mL quartz cuvette, mix rapidly and then compare the colour at 234 nm, record the absorbance values at 15 s and 75 s, which were recorded as A1 and A2, respectively. Measurement tube: add 100 μL of supernatant, 800 μL of reagent 1 and 100 μL of reagent 2 into 1 mL quartz cuvette, mix quickly and then compare the colour at 234 nm. After rapid mixing, the colour was measured at 234 nm and the absorbance values at 15 s and 75 s were recorded as A3 and A4 respectively.

In a system of 1 mL, a change of 0.01 units per minute in catalytic absorbance value per gram of sample in 25 °C is one unit of enzyme activity.

LOX (U/g) = [(A4-A3)-(A2-A1)]) × total V counter ÷ (W × total V sample ÷ total V sample) = 33.33 × ΔA ÷ W.

Total V counter: total volume of reaction, 1 ml;

total V sample: total volume of supernatant, 1 ml;

W is the mass of the sample, g.

Section S6. Lipidomics analysis (Chromatographic conditions and elution procedures):

Positive source: mobile phase A: water: methanol: acetonitrile (7 mM ammonium acetate, 1 : 1 : 1, v : v : v); mobile phase B: isopropanol (7 mM ammonium acetate); flow rate 0.3 mL /min; injection volume 1 μL; column temperature 45 °C. The sample was extracted from the sample at a flow rate of 0.5 mL/min.

Positive source gradient elution procedure: 0-1.5 min, maintain 20% B, 1.5-3 min, gradient increase to 40% B, 3-13 min, gradient increase to 60% B, 13-13.1 min, increase to 98% B, 13.1-17 min, decrease to 20% B.

Negative source: mobile phase A: isopropanol: acetonitrile (5 mM ammonium acetate, 7 : 93, v : v); mobile phase B: water: acetonitrile (2 mM ammonium acetate, 1:1, v : v); injection volume: 1 μL; column temperature: 35 °C. The sample was taken at a flow rate of 1.5 μL.

Negative source gradient elution procedure: 0-2 min, maintain 0% B, 2.01-7 min, gradient increases to 50% B, 11-11.5 min, gradient increases to 70%, 11.5-12.5 min, gradient increases to 100% B, 12.5-15 min, gradient maintains 100% B, 15-15.1 min, gradient decreases to 0% B, the 15.1-17 min, maintaining 0% B. Flow rate 0-2 min: 0.2 mL/min, 2.01-15 min: 0.7 mL/min, 15.01-17 min: 0.2 mL/min.

Section S7. Metabolomics analysis (Chromatographic conditions and elution procedures):

Positive Source: Mobile phase A: 0.1 % formic acid-water; Mobile phase B: 0.1 % formic acid-acetonitrile. The flow rate was 0.35 mL/min, injection volume was 1 μL, and the column temperature was 40 °C. The sample was extracted at a flow rate of 0.35 mL/min and the injection volume was 1 μL.

Positive source elution procedure: 0 min, 95 % A, 5 % B; 1 min, 95 % A, 5 % B; 24 min, 0 % A, 100 % B; 28 min, 0 % A, 100 % B; 28.1 min, 95 % A, 5 % B; 30 min, 95 % A, 5 % B.

Negative sources: Mobile phase A: 0.1% ammonia-water; Mobile phase B: 0.1% ammonia-95% methanol-water.

Negative source elution procedure: 0 min, 98 % A, 2 % B; 1 min, 98 % A, 2 % B; 18 min, 0 % A, 100 % B; 22 min, 0 % A, 100 % B; 22.1 min, 95 % A, 5 % B; 25 min, 95 % A, 5 % B. Flow rate: 0.35 mL/min; injection volume: 1 μL; column temperature: 40 ℃.

Section S8. Determination of glucose, fructose, sucrose (Chromatographic methods):

Mobile phase: acetonitrile : water=7 : 3 (v : v); flow rate: 1.0 mL/min; column temperature: 40 ℃; injection volume: 20 μL; detector: 2414 oscillometric refractive detector, detection cell temperature: 35 ℃.

Section S9. Some DIMs in the Control, CI and CTS treatments.

	Glutamic acid	Arginine	Spermine	Isoleucine	α,α-Trehalose	Pyruvate	Oxaloacetate	Fumarate	Succinate	Mesaconate
**Control-1**	1.56E+05	1.18E+06	2.58E+07	8.24E+07	3.56E+07	6.34E+08	8.85E+07	2.12E+04	8.99E+07	1.31E+08
**Control-2**	1.56E+05	1.14E+06	2.18E+07	8.23E+07	3.01E+07	5.67E+08	8.85E+07	2.12E+04	8.99E+07	1.31E+08
**Control-3**	1.79E+05	1.06E+06	2.07E+07	8.47E+07	3.06E+07	5.67E+08	8.24E+07	2.45E+04	8.78E+07	1.16E+08
**Control-4**	1.68E+05	1.09E+06	2.21E+07	8.82E+08	3.28E+07	6.27E+08	8.24E+07	2.20E+04	8.78E+07	1.27E+08
**Control-5**	1.68E+05	1.26E+06	2.14E+07	8.14E+07	3.28E+07	7.27E+08	7.75E+07	3.23E+04	8.95E+07	1.13E+08
**Control-6**	1.40E+04	1.25E+06	2.14E+07	8.14E+07	3.01E+07	5.97E+08	7.95E+07	2.94E+04	8.96E+07	1.11E+08
**CI-1**	8.81E+07	1.42E+07	2.39E+08	1.34E+09	6.02E+08	5.40E+09	1.77E+08	8.99E+05	2.40E+08	7.45E+08
**CI-2**	8.60E+07	1.26E+07	1.73E+08	1.25E+09	6.19E+08	5.11E+09	1.82E+08	7.20E+05	2.62E+08	7.17E+08
**CI-3**	8.38E+07	1.09E+07	1.83E+08	1.43E+09	6.11E+08	5.11E+09	1.79E+08	8.00E+05	2.67E+08	7.23E+08
**CI-4**	8.81E+07	1.16E+07	2.00E+08	1.34E+09	5.86E+08	4.97E+09	1.77E+08	7.89E+05	2.49E+08	7.21E+08
**CI-5**	8.36E+07	1.36E+07	1.91E+08	1.25E+09	6.18E+08	4.96E+09	1.73E+08	8.39E+05	2.18E+08	7.41E+08
**CI-6**	8.63E+07	1.26E+07	2.00E+08	1.43E+09	6.31E+08	5.73E+09	1.80E+08	7.93E+05	2.38E+08	7.51E+08
**CTS-1**	1.92E+08	4.36E+07	2.06E+08	5.32E+09	1.29E+10	1.10E+10	4.57E+08	1.09E+05	5.26E+08	1.31E+09
**CTS-2**	1.94E+08	4.87E+07	2.11E+08	5.23E+09	1.28E+10	1.20E+10	4.66E+08	1.26E+06	5.79E+08	1.34E+09
**CTS-3**	1.82E+08	5.01E+07	1.99E+08	5.13E+09	1.19E+10	1.17E+10	4.43E+08	1.10E+06	5.57E+08	1.38E+09
**CTS-4**	1.95E+08	5.22E+07	1.96E+08	5.07E+09	1.21E+10	1.19E+10	4.23E+08	1.16E+06	5.67E+08	1.19E+09
**CTS-5**	1.91E+08	5.39E+07	2.00E+08	5.57E+09	1.31E+10	1.18E+10	4.38E+08	1.32E+06	5.56E+08	1.23E+09
**CTS-6**	1.98E+08	4.81E+07	2.21E+08	5.03E+09	1.21E+10	1.17E+10	4.56E+08	1.42E+06	5.85E+08	1.38E+09

Note: The treatments were as follows: Control (soaked in distilled water and stored at 25 °C for 10 days); CI (soaked in distilled water, stored at 4 °C for 4 days, followed by storage at 25 °C for 6 days); CTS (soaked in 1% CTS solution, stored at 4 °C for 4 days, followed by storage at 25 °C for 6 days).

References

1. Yang, W. H. Study on the control effect of five exogenous substances on chilling injury in postharvest banana fruit. Master's thesis, *Guangxi University*, **2021**. https://doi:10.27034/d.cnki.ggxiu.2021.000189 (In Chinese)
